# Integrated Mobile Element Scanning (ME-Scan) method for identifying multiple types of polymorphic mobile element insertions

**DOI:** 10.1186/s13100-020-00207-x

**Published:** 2020-02-22

**Authors:** Jui Wan Loh, Hongseok Ha, Timothy Lin, Nawei Sun, Kathleen H. Burns, Jinchuan Xing

**Affiliations:** 1 Department of Genetics, Rutgers, the State University of New Jersey, Piscataway, NJ 08854 USA; 2 Human Genetic Institute of New Jersey, Rutgers, the State University of New Jersey, Piscataway, 08854 NJ USA; 3 Department of Pathology, Johns Hopkins University School of Medicine, Baltimore, 21205 MD USA

**Keywords:** *Alu*Yb, SVA, LINE-1, Retrotransposon, Mobile element insertion, High-throughput sequencing, ME-Scan

## Abstract

**Background:**

Mobile elements are ubiquitous components of mammalian genomes and constitute more than half of the human genome. Polymorphic mobile element insertions (pMEIs) are a major source of human genomic variation and are gaining research interest because of their involvement in gene expression regulation, genome integrity, and disease.

**Results:**

Building on our previous Mobile Element Scanning (ME-Scan) protocols, we developed an integrated ME-Scan protocol to identify three major active families of human mobile elements, *Alu*Yb, L1HS, and SVA. This approach selectively amplifies insertion sites of currently active retrotransposons for Illumina sequencing. By pooling the libraries together, we can identify pMEIs from all three mobile element families in one sequencing run. To demonstrate the utility of the new ME-Scan protocol, we sequenced 12 human parent-offspring trios. Our results showed high sensitivity (> 90%) and accuracy (> 95%) of the protocol for identifying pMEIs in the human genome. In addition, we also tested the feasibility of identifying somatic insertions using the protocol.

**Conclusions:**

The integrated ME-Scan protocol is a cost-effective way to identify novel pMEIs in the human genome. In addition, by developing the protocol to detect three mobile element families, we demonstrate the flexibility of the ME-Scan protocol. We present instructions for the library design, a sequencing protocol, and a computational pipeline for downstream analyses as a complete framework that will allow researchers to easily adapt the ME-Scan protocol to their own projects in other genomes.

## Background

Mobile genetic elements, also known as transposable elements, are a major component of mammalian genomes and account for more than half of the human genome [1, 2]. In the human genome, retrotransposons are the only class of mobile elements that are still actively propagating. Specifically, three families of non-Long Terminal Repeat (non-LTR) retrotransposons account for the vast majority of human-specific mobile element insertions (MEIs): the *Alu* element, the long interspersed element 1 (LINE-1 or L1), and the composite SINE-R/VNTR/Alu (SVA) element [3–5]. Some of these insertions happened recently in humans and are still present as polymorphic sites among human populations [4, 6, 7]. These polymorphic MEIs (pMEIs) contribute to human genomic diversity, as well as genome function. pMEIs have been shown to regulate gene expression [8, 9], to “exonize” into protein coding sequences [10–12], as well as to cause a variety of human diseases [13–15]. Retrotransposon expression has also been associated with different types of cancer [16–18], and neurological disorders [19, 20]. For these reasons, it is important to understand the distribution and prevalence of pMEIs in human populations.

The development of high-throughput sequencing technology drastically improves our ability to identify and characterize pMEIs (Reviewed in [21–23]). One approach is to identify pMEIs from whole-genome sequencing (WGS) data [24, 25]. Although high-coverage WGS is suitable for studying MEs in different species, WGS of mammalian genomes at the population scale is still expensive and computational methods detecting pMEIs from WGS data usually suffer from low specificity and high false-positive rate [23, 24, 26]. To overcome these limitations, target-enrichment methods can be used to construct MEI-specific sequencing libraries for studying specific types of MEIs. Developed in the past few years, these methods included both PCR-based and probe-based enrichment strategies (Reviewed in [21]). PCR-based enrichment methods usually use a pair of primers to amplify the ME/genomic junction site: one primer that is specific to an ME of interest, and the 2nd primer that either binds to a generic linker sequence or to random genomic sequences [27–33]. The PCR-based methods have also been used lately with a multiplex modification [33, 34]. In contrast, the probe-based enrichment methods typically use ME-specific probes to enrich DNA fragments containing one of several types of MEs from the genomic DNA [35–37]. Although the earlier probe-based methods have relatively low specificity, more recent methods have been improved by the use of chemically modified probes such as Locked Nucleic Acid [33, 38].

Despite the advantage of low cost and high specificity, PCR-based methods usually focus on one specific type of ME [27–33]. To address this issue, we developed an integrated Mobile Element Scanning (ME-Scan) protocol building upon our previous ME-Scan protocols [28, 29, 39, 40]. This integrated protocol allows simultaneous sequencing and characterization of three major active families of human mobile elements, *Alu*Yb, L1HS, and SVA. By pooling sequencing libraries together, we can identify pMEIs from all three ME families in one sequencing run. In addition to the improved molecular protocol, we also provide a computational pipeline for the data analysis. This method is a cost-effective way to identify MEIs for both large-scale genomic studies and transposon-based mutagenesis studies. In this study, we demonstrate the utility of this protocol by applying the protocol to 12 human parent-offspring trios. We also apply the protocol to four different cell types from three samples to test the feasibility of identifying somatic pMEIs in different cell types.

## Results

### Protocol overview

**Fig. 1:**
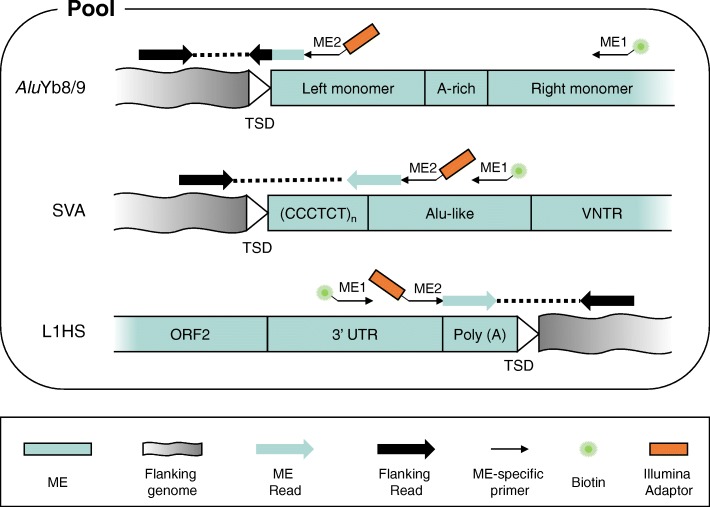
ME-specific amplification during ME-Scan library construction. For each ME type library, two rounds of nested amplification are performed. The ME-specific amplification primers (ME1 and ME2) are shown as thin arrows above the ME consensus and the amplification directions are indicated by the arrows. First-round amplification primers (ME1) are biotinylated (green star) for enrichment, and the second-round nested primers (ME2) include the Illumina sequencing adaptor (orange box). Different components of *Alu*Yb, SVA, and L1HS consensuses are labelled. The final paired-end sequencing reads from the resulting sequencing libraries are represented with blue arrows (ME Reads) and black arrows (Flanking Reads), respectively. Blue box: ME sequence; grey box: flanking genomic region; green star: biotin; orange box: Illumina sequencing adaptor

We previously described the ME-Scan protocol with two rounds of nested PCRs for *Alu*Yb8/9 elements (referred to as *Alu*Yb in the following text) [28, 29] and full-length SVA elements [40]. In this study, we extended our protocol to L1HS elements to cover all three main active retrotransposon families in the human genome. The ME-Scan *Alu*Yb and SVA protocols enrich for the ME/flanking genome junction at the 5′ end of the MEs (Fig. 1). In contrast, the ME-Scan L1HS protocol targets the 3′ end of the insertion (Fig. 1). This allows us to exploit the internal 3’UTR sequence variants that are unique to the active, species-specific L1HS subfamily, to cover the insertion site with short amplicon lengths, and to robustly recover L1 elements with 5′ end truncation. The diagnostic 3′ nucleotides of L1HS was shown to vastly increase the specificity of targeted libraries [27], and similar primer-design strategy has been used in several L1HS-enrichment protocols [27, 31, 32, 41].

**Fig. 2:**
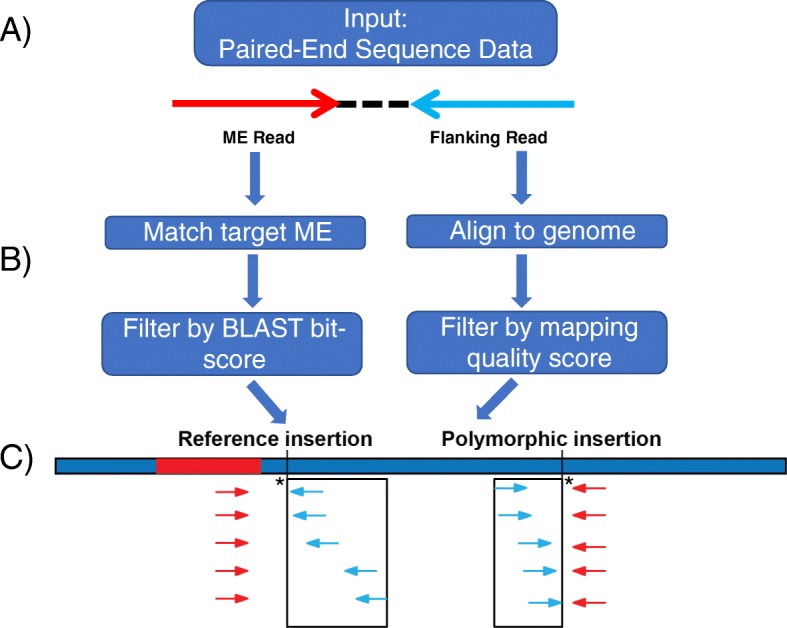
Computational data analysis overview. **a)** The paired-end sequencing reads. Sequencing reads from the pooled libraries are represented by red (ME Reads) and blue arrows (Flanking Reads), respectively. **b)** Read filtering. The ME Reads are compared to the targeted ME consensus to identify recent insertions and are filtered based on the BLAST bit-score cutoff. The Flanking Reads are mapped to the reference genome and are filtered based on the mapping quality score cutoff. **c)** Flanking Read clustering and insertion loci identification. Filtered Flanking reads that are within a 500 bp sliding window are clustered into a candidate insertion locus and the genomic position closest to the ME Read is selected as the insertion position (marked with a star). Black box: clustering window

To facilitate the analysis of the data from the combined libraries, we establish a ME-Scan computational analysis pipeline that can be used to analyze sequencing data from different types of MEs. Figure 2 shows a simplified outline of the analysis steps. A detailed pipeline is described in the method section and in Figure S1. The computational method relies on the primer design and inherent properties of the sequenced reads. Briefly, using the Illumina pair-end sequencing format, two sequencing reads are generated from each DNA fragment encompassing a specific ME (Fig. 2a). Read 1 contains the ME sequences (red read in Fig. 2, referred as the **ME Read** in the following text) and is used to determine if a read-pair is derived from a targeted ME family. The second read in the read-pair, Read 2, lies outside of the ME region (blue read in Fig. 2, referred as the **Flanking Read** in the following text) and is aligned to the reference genome using Burrows-Wheeler Aligner (BWA) to identify the genomic location of an MEI. Both ME Read and Flanking Read need to be filtered to improve the accuracy of the identified candidate loci (Fig. 2b).

To filter the ME Read, we first establish an ME-specific BLAST bit-score cutoff for each ME family based on the insertions in the human reference genome (Figure S2). The cutoff is selected to enrich for the targeted ME. For example, for L1HS we select a cutoff of 56 to ensure the vast majority of ME Reads are from the L1HS subfamily. The ME Reads are then filtered by the BLAST bit-score cutoff to select loci containing recent and potential polymorphic MEIs. Flanking Reads, on the other hand, are filtered based on their mapping quality scores (MQ) to ensure the high-confidence mapping of the reads (Fig. 2b). The MQ filtering is crucial for Flanking Reads that are from repetitive genomic regions and can be mapped to multiple genomic locations. For reads that can be mapped perfectly to multiple genomic locations, one of the mapping positions is reported in the BWA output. If different sections of a read can be mapped to different genomic locations, multiple positions could be reported in the BWA output. Our pipeline filters out most of these multiple mapping reads in two steps: Step 1, if multiple mapping positions are reported for a read, only one position with the highest MQ is selected. This filter ensures that each read is only present once in the mapping result. Step 2, we apply a stringent mapping quality filtering (MQ > =30) to the BWA output. Reads that are mapped perfectly to multiple genomic locations have an MQ of 0 and therefore are excluded from the downstream analysis after the MQ filtering. For reads that can be partially mapped to multiple locations, the vast majority of the mapping positions have low MQs and were excluded from the analysis. Only a small fraction of multiple partial mapping reads has MQ > =30 and is included in the downstream analysis.

Next, the end positions of the mapped and filtered Flanking Reads that are on the same strand are sorted and clustered within a sliding window of 500 base pairs (bps) in size to define putative MEI loci (Fig. 2c). Within each cluster, the Flanking Read mapping position that is the closest to the ME Read is chosen as the insertion position for that MEI locus (Fig. 2c, stars). To assess the support of each putative pMEI locus, we calculate two evidence metrics for the Flanking Reads in each cluster. First, we count the number of mapped Flanking Reads and normalize the count by the total number of mapped reads in each individual (TPM, tags per million). This normalization accounts for inter-library variation. Second, we count the number of uniquely mapped Flanking Reads in the window for each individual (UR, unique reads). Using the combination of TPM and UR information for each locus, we calculate the sensitivity for identifying fixed MEIs under different TPM and UR cutoffs. We determine individual-specific TPM and UR cutoffs as the highest TPM and UR combination (with a maximum value of 10 TPMs and 10 URs) that allows for the identification of more than 90% of the presumably fixed reference MEIs (See Methods for details). This way we control the sensitivity of our assay by its ability to identify known fixed insertion sites. We showed previously that the combination of TPM and UR cutoffs provide a good quality assessment for identifying MEI loci [40]. Once all potential MEI loci are identified, the loci are then compared to the reference genome and to the known polymorphic loci to annotate the candidate loci as known and novel pMEIs, respectively (see Methods for details).

### Applying ME-scan to population samples

To demonstrate the utility of the integrated ME-Scan protocol, we applied the method to 36 samples from 12 parent-offspring trios from the HapMap population Yoruba in Ibadan, Nigeria (YRI). The sequencing depth and the number of reads that passed filter for *Alu*Yb, L1HS, and SVA in each sample are shown in Table S1. Overall, 188, 183, and 256 million read pairs were obtained from the *Alu*Yb, L1HS, and SVA ME-Scan libraries, respectively. To enrich for recent MEIs, we applied BLAST bit-score cutoffs of 67, 56, and 48, for *Alu*Yb, L1HS, and SVA ME Reads, respectively. After filtering the Flanking Reads with a mapping quality score cutoff of 30, we clustered Flanking Reads in 500 bps sliding windows to define putative MEI loci. For each putative MEI locus, we calculated the TPMs and URs cutoffs that allow for the identification of more than 90% of the presumably fixed reference MEIs, as described in the protocol overview section. After applying cutoffs that were tuned for each ME type in each individual (Table 1), 4216 *Alu*Yb, 2250 L1HS, and 1779 SVA elements were identified from the 36 individuals. Among them, 1819 *Alu*Yb, 1456 L1HS, and 477 SVAs were polymorphic among the individuals, and 1079 *Alu*Yb, 1175 L1HS, and 180 SVAs appeared to be novel to this study (Table 1).

**Table 1:** Cutoffs and the number of candidate loci in YRI individuals

	*Alu*Yb				L1HS				SVA			
Individual	Cutoff (TPM,UR)	All	Poly-morphic	Novel	Cutoff (TPM,UR)	All	Poly-morphic	Novel	Cutoff (TPM,UR)	All	Poly-morphic	Novel
NA18500	(10,10)	2411	387	168	(10,10)	951	234	108	(4,9)	1395	164	13
NA18501	(10,10)	2453	416	188	(10,10)	949	231	112	(3,10)	1398	178	12
NA18502	(10,10)	2546	452	206	(10,10)	968	252	142	(5,10)	1393	172	14
NA18503	(10,10)	2418	392	181	(10,10)	950	237	126	(4,10)	1390	166	10
NA18504	(10,10)	2463	418	191	(10,10)	981	257	133	(3,9)	1400	170	7
NA18505	(10,10)	2494	427	183	(10,10)	1028	301	159	(4,10)	1407	175	9
NA18506	(10,8)	2347	362	139	(10,10)	916	211	116	(4,6)	1392	170	15
NA18507	(10,10)	2408	383	145	(10,10)	922	220	115	(3,10)	1404	174	11
NA18508	(10,10)	2634	509	242	(10,10)	998	276	156	(5,10)	1405	175	15
NA18515	(10,10)	2563	445	213	(10,10)	982	255	135	(5,10)	1393	162	13
NA18516	(10,10)	2554	448	208	(10,10)	1049	310	172	(6,10)	1369	141	7
NA18517	(10,10)	2566	464	224	(10,10)	1023	293	165	(4,10)	1417	183	21
NA18521	(10,10)	2572	470	218	(10,10)	959	238	131	(5,10)	1398	163	14
NA18522	(10,10)	2562	449	205	(10,10)	967	237	122	(5,10)	1402	171	14
NA18523	(10,10)	2689	533	252	(10,10)	1047	319	176	(5,10)	1427	193	23
NA19101	(10,10)	2534	424	184	(10,10)	1001	273	147	(5,10)	1378	154	5
NA19102	(10,10)	2550	445	193	(10,10)	1018	286	157	(6,10)	1381	149	4
NA19103	(10,10)	2455	398	161	(10,10)	1005	278	155	(5,7)	1372	149	8
NA19137	(10,10)	2593	476	201	(10,10)	995	268	144	(6,10)	1379	150	10
NA19138	(10,10)	2604	467	201	(10,10)	1040	299	156	(7,10)	1376	146	11
NA19139	(10,10)	2619	480	202	(10,10)	1021	285	152	(7,10)	1370	147	11
NA19171	(10,10)	2652	503	219	(10,10)	1085	333	188	(6,10)	1374	149	6
NA19172	(10,10)	2668	533	231	(10,10)	1100	350	194	(7,10)	1381	149	11
NA19173	(10,10)	2564	438	173	(10,10)	1035	292	160	(7,10)	1361	138	9
NA19200	(10,10)	2617	479	209	(10,10)	1029	293	163	(6,10)	1387	158	8
NA19201	(10,10)	2567	455	199	(10,10)	1004	275	151	(7,10)	1367	140	7
NA19202	(10,10)	2669	510	230	(10,10)	1098	358	204	(6,10)	1384	155	10
NA19203	(10,10)	2559	433	179	(10,10)	1051	307	165	(6,10)	1374	151	4
NA19204	(10,10)	2686	534	239	(10,10)	1160	408	254	(7,10)	1378	152	14
NA19205	(10,10)	2589	454	199	(10,10)	1024	290	158	(6,10)	1368	145	9
NA19206	(10,10)	2270	349	122	(10,9)	953	248	129	(4,4)	1392	172	13
NA19207	(10,10)	2516	422	184	(10,10)	1062	325	191	(6,10)	1371	140	10
NA19208	(10,10)	2491	410	169	(10,10)	1011	278	144	(4,9)	1381	152	8
NA19209	(10,10)	2544	432	195	(10,10)	1025	296	160	(7,10)	1367	141	8
NA19210	(10,10)	2615	473	198	(10,10)	1073	336	197	(6,10)	1380	147	11
NA19211	(10,10)	2485	412	176	(10,10)	1037	296	161	(5,10)	1375	150	3
Total		4216	1819	1079		2250	1456	1175		1779	477	180

**Fig. 3:**
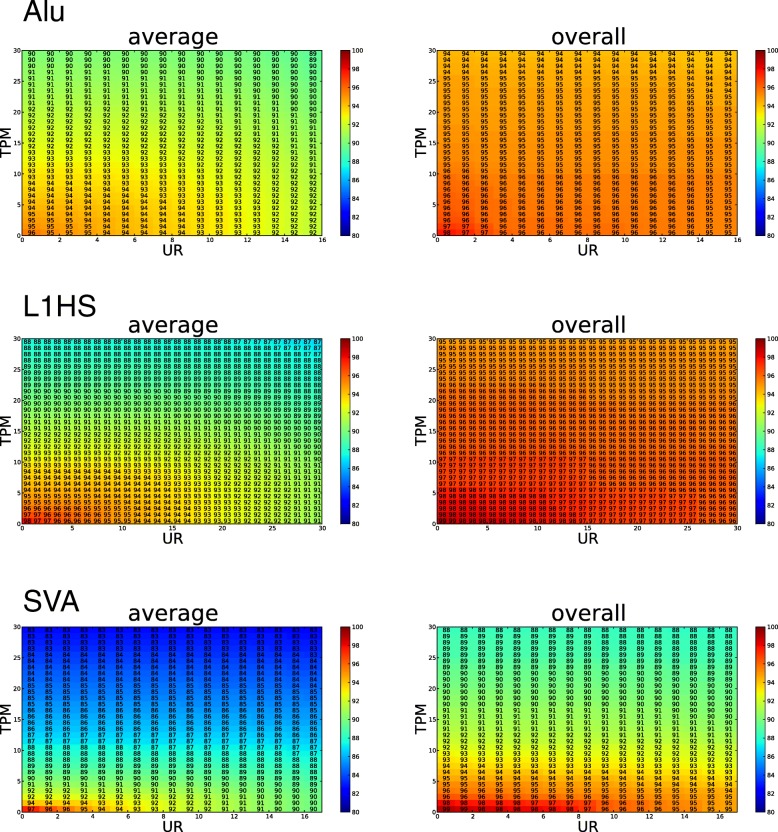
Sensitivity analysis for determining proper TPM and UR cutoffs. Using presumably fixed reference MEIs as true positives, the sensitivity is calculated under different TPM and UR cutoffs for *Alu*Yb, L1HS, and SVA candidate loci, respectively. The average individual sensitivity (left panel) and overall sensitivity (right panel) for the 36 YRI samples are shown. The sensitivity is shown as the percentage of presumably fixed insertions being identified for each cutoff. The heatmap color corresponds to the sensitivity, as indicated in the color bar on the right of each plot

At the selected TPM/UR cutoffs (~ 10/10 for *Alu*Yb and L1HS, ~ 5/10 for SVA), all three libraries showed high sensitivity for the presumably fixed elements in the reference genome: *Alu*Yb and L1HS have a comparable average individual sensitivity of 93%, while SVA has a 90% average individual sensitivity (Fig. 3, “average”). The overall sensitivity of our methodology to identify fixed reference elements is above 95% for all three retrotransposon families when all individuals were combined (Fig. 3, “overall”). This high sensitivity suggests that with the pooled ME-Scan libraries we can recover most of the polymorphic elements in the targeted ME families.

Next, we assessed the accuracy of our pMEI calls using the parent-offspring trio information. An MEI that is found in a child but not in either of the parents does not fit the expected inheritance pattern. The MEI can be an authentic *de novo* insertion in the child, a false-positive call in the child, or false-negative calls in the parents. Because any insertion that is present in multiple individuals are unlikely to be a *de novo* insertion, we define a *de novo* insertion candidate as an insertion that is present in a child and absent in all other 35 individuals among the 12 trios. In total, 5 L1HS and 19 SVA *de novo* candidates were identified. Given the low retrotransposition rate for human retrotransposons (< 1 in 10 live births for *Alu*, < 1 in 100 live births for L1HS and SVA), we did not expect to identify any *de novo* L1HS or SVA insertions in 12 trios. Indeed, upon a close inspection we observed that nearly all candidate loci are in the vicinity of old retrotransposons or repetitive regions in the reference genome (Table S2). In general, the supporting Flanking Reads have low mapping quality because of the repetitive nature of these regions. Consistent with this observation, several *de novo* insertion candidates that we attempted to validate failed to amplify the expected insertion sites (data not shown). Therefore, these loci are more likely to be either sequencing or mapping artifacts and we did not validate any authentic *de novo* insertions. Assuming that all of these *de novo* candidates are false calls, the inheritance error rates for the pMEIs are still low: the average inheritance error rates for the 12 trios are 0.33, 4.96, and 2.23% for *Alu*Yb, L1HS, and SVA, respectively (Table S3). These low inheritance error rates suggest that the vast majority of the pMEIs we identified are real insertions.

Using the trio information, we also assessed the false-negative rate in our dataset. To reduce the effect of false-positive calls in the parents, for the analysis we selected pMEIs that are present in at least two individuals among the 24 parents. For a locus where the pMEI is present in only one parent, the expected inheritance rate of the locus is either 50% (heterozygous insertion in the parent) or 100% (homozygous insertion in the parent). The average observed inheritance rate among the 12 trios are about 52% for *Alu*Yb, L1HS, and SVA elements, ranging from 42 to 65% (Table S4). One possible reason for the inheritance rate close to 50% is most of the insertions are rare and are present as heterozygous in the parent. If we assume the highest inheritance rate (65%) in the family Y045 is the true inheritance rate, on average other trios have a false-negative rate around 15%. For a locus where the pMEI is present in both parents, the expected inheritance rate of a locus is either 75% (heterozygous in both parents) or 100% (one or both parents are homozygous). The average observed inheritance rate among the 12 trios are 86, 87, and 89% for *Alu*Yb, L1HS, and SVA elements, respectively (ranging from 80 to 95%, Table S4). Similar to the single-parent loci, Y045 has the highest inheritance rate of 92, 94, and 95% for *Alu*Yb, L1HS, and SVA elements, respectively. pMEIs present in both parents of a trio are expected to be more common in the population than the single-parent loci. As expected, the inheritance rate is closer to the high end of the expectation (100%) than the low end (75%). If we assume the highest inheritance rate in the family Y045 is the true inheritance rate, on average other trios have a false-negative rate around 5, 7, and 6% for *Alu*Yb, L1HS, and SVA elements, respectively.

Lastly, we determined the functional impact of pMEIs. Similar to previous studies, the vast majority of the pMEIs were non-exonic (Figure S3A). Among the polymorphic MEIs, 13 overlapped coding sequence (CDS), including two *Alu*Yb, eight L1HS, and three SVA insertions (Table S5). Of those, 10 have not been previously reported and are novel pMEIs. We were able to validate the novel *Alu*Yb insertion (Alu_CDS1) with locus-specific PCR and Sanger sequencing (Figure S4A, S4C). Among the six novel L1HS insertion loci where primers can be designed, we successfully confirmed the targeted junction from the original genomic DNA sample for four loci (Table S5, Figure S4B). However, Sanger sequencing of the entire loci containing the L1HS insertion will be needed to formally validate these loci. Examining the chromatin states of the pMEI locations revealed that most of the insertions are in chromatin state 13 (Heterochromatin; low signal) [42], suggesting they are not involved in active transcription (Figure S3B).

### Searching for somatic insertions during iPSC induction and cell differentiation

In recent years, it has been recognized that retrotransposition activities are not limited to the germline. Instead, somatic MEIs were shown to exist in different tissues (Reviewed in [43]). To test if the ME-Scan protocol can be used to identify somatic MEIs, we obtained DNA samples from three individuals, a mother and her two offspring [44]. For each individual, DNA samples from four cell types were collected, including CD4^+^ T lymphocytes, induced pluripotent stem cells (iPSCs) generated from the CD4^+^ T lymphocytes, neural stem cells (NSCs) derived from the iPSCs, and neurons differentiated from the NSCs. We constructed *Alu*Yb, L1HS, and SVA ME-Scan libraries for each of the four cell types for the three individuals and pooled all libraries in one sequencing run. Overall, 9.8, 96, and 117 million mapped read pairs were obtained from the *Alu*Yb, L1HS, and SVA ME-Scan libraries, respectively. The sequencing depth and the number of reads that passed filter for *Alu*Yb, L1HS, and SVA in each sample are shown in Table S6.

We first identified all non-reference MEI loci among the 12 samples using the same computational pipeline for the population samples. In total, there are ~ 250 *Alu*Yb, ~ 210 L1HS, and ~ 170 SVA elements that are present in all four cell types in each individual (Additional file 2). These are likely germline insertions and the number of insertions in each individual is comparable to the number of pMEIs in population samples (Table 1). To identify somatic insertion candidates, we excluded loci that are known pMEIs, and loci that have reads from multiple individuals. Within each individual, a locus is defined as cell-type specific if all other cell types have zero reads. After filtering, there was no *Alu*Yb and L1HS somatic insertion candidates. Seven SVA somatic candidate loci were identified in three different cell types (Table S7). Upon a close inspection, all seven candidate loci are either inside of old *Alu*Yb elements or repetitive regions in the reference genome (Table S7). Therefore, these insertions are likely to be false-positives. We attempted to validate two neuron-specific SVA insertions by a locus-specific three-primer PCR strategy [6, 40]. We were unable to generate specific amplification product to validate the loci. The small number of candidate loci and the failed validation suggest that somatic MEIs are rare; higher sequencing coverages and larger sample sizes would be needed for the ME-Scan protocol to accurately identify somatic insertions.

## Discussion

In this study, we presented a framework for using the ME-Scan protocol to detect multiple types of ME in a single sequencing experiment set up. We presented both the protocol for library construction and the downstream computational analysis pipeline. To demonstrate the utility of the protocol, we applied the protocol to three major active human ME families, *Alu*Yb, L1HS, and SVA. We demonstrated high sensitivity and specificity for identifying germline pMEIs. The number of polymorphic *Alu*Yb and SVA elements identified in our population samples was smaller than previous studies because of the protocol design: the current *Alu* protocol is designed to capture one of the major polymorphic subfamilies: *Alu*Yb. Therefore, polymorphic *Alu*Ya and *Alu*Y elements will not be identified by the current protocol. Similarly, the SVA protocol is designed to identify full-length insertions with intact 5′ end. Therefore, SVA elements with 5′ truncation are not present in our library.

In addition to germline insertions, we also attempted to identify somatic insertions. Previous studies showed that somatic mobile element insertions can happen during the iPSC conversion [36, 45, 46] and during neuronal differentiation [34, 43, 47–50]. Therefore, we carried out ME-Scan protocol in T cells, iPSC, NSC, and neurons in three individuals. Although candidate somatic pMEIs could be identified, we were not able to validate any of the somatic insertion candidates using locus-specific PCR. Inability to identify and to validate the somatic insertion sites could be explained by several reasons. First, the somatic insertions are rare in cell populations and the sequencing depth in our experiment does not have sufficient power to detect somatic insertions from DNA extracted from a large batch of cells. For example, Salvador-Palomeque et al. identified one *de novo* L1 insertion in a human iPSC cell line using the probe-based RC-Seq approach [46]. The number of sequencing reads per sample ranges from 24 to 64 million in the study. In contrast, our L1HS pass-filter reads ranges from 1.7 to 10 million per sample (average 5.7 million, Table S6). Therefore, our sequencing depth was several folds lower than the Salvador-Palomeque et al. study. Second, because the *de novo* insertions could be present in only a small percentage of the cells, the locus-specific PCR validation needs further modifications from established protocols. Indeed, validating somatic pMEIs, especially with internal ME primers, is known to be difficult [32]. In the future, it would be informative to test the ME-Scan protocol on tumor samples that have been tested previously and have shown to have high rate of somatic insertions. This experiment would allow us to determine the sensitivity of the protocol and the necessary coverage for somatic insertion identification. Recently, many methods have also been developed to specifically target somatic MEIs at the single cell level [34, 38, 46, 49–52], including probe-based single-cell RC-seq methods [38, 46]. These methods might be better suited for somatic insertions validations in the future.

In the current form, ME-Scan protocol has some limitations. First, the protocol is based on the Illumina sequencing technology. Similar to other next-generation sequencing technologies, Illumina produces short sequencing reads (100 bps in our case). These short reads make it difficult to identify MEIs in highly repetitive genomic regions. In our pipeline we exclude most reads that can be mapped to multiple locations to reduce false-positive calls, and in the process some of the real MEIs in the repetitive regions may have been lost. In addition, L1 and SVA insertions are known to contain additional genomic sequences from run-through transcripts, a process termed transduction [10, 53]. Our L1HS protocol amplifies the 3′ end of the L1HS elements, and the amplicons are likely to contain the transduced sequence. Whether an L1 insertion with 3′ transduction can be detected depending on the size of the transduction. Our current protocol selects DNA fragments that are 500–1000 bp in size. If a transduction is small (e.g., 100-200 bp), there will be enough flanking genomic sequence at the insertion site for identifying the new insertion locus. However, if a transduction is larger than the fragment size, Flanking Reads will be within the transduction sequence and are likely to be mapped to the original genomic position. Other amplification-based L1 identification method (e.g. L1-IP) has been shown to have similar limitations on L1 insertions with long 3′ transduction [50]. Probe-based method (e.g. RC-Seq) or WGS-based approach can provide better sensitivity to insertions with transductions [50]. Recently, long-read, single-molecule sequencing technologies (e.g. Pacific Biosciences and Oxford Nanopore) are becoming more practical. Incorporating long-read sequencing technology into the ME-Scan protocol can improve the identification of MEIs in repetitive regions and MEIs with transductions. Second, as a PCR-based protocol, the amplification step could introduce locus-specific biases and miss MEIs that acquired mutations, especially insertion and deletion, at the primer binding sites. This issue should not be a concern when the MEIs of interest are recent insertions and have high similarity to the consensus sequence. If a researcher needs to study old or highly diverged pMEIs, multiple primers can be designed across the mobile element to improve the sensitivity. In fact, we applied a mixture of three L1HS primers during the first round of amplification to allow variations at the amplification site (Table 2). Third, the current protocol captures only one side of the mobile element flanking sequence and does not provide a full picture of the insertion site. Nevertheless, as we demonstrated with the *Alu*Yb, L1HS, and SVA primer designs, the protocol can be used to extend towards both the 5′ end (*Alu*Yb and SVA) and the 3′ end (L1HS) of the MEs. If obtaining both sides of the flanking sequence is crucial for an application and the MEIs do not contain extensive truncations, the researcher can design primers for amplifying both ends of the MEI consensus. For elements that are often truncated (e.g., 5′ of L1 insertions), probe-based enrichment or WGS-based method might be a better option. Comparing to the probe-based enrichment or WGS-based method, the main advantage of amplification-based methods is the high specificity, which allows a much higher coverage on the targeted elements at lower cost. For example, the cost saving for L1HS can be more than 100 folds when compared to WGS-based method [32].

**Table 2:** Oligos and primers used in this study

Description	Sequence (5′- > 3′)
Long adaptor with indexes	CAAGCAGAAGACGGCATACGAGAT***Index1***GTGACTGGAGTTCAGACGTGTGCTCTTCCGATCTIndex2*T
Short adaptor with indexes	***Index2***AGATCGGAAGAGCGTCGTG
1st round *Alu*Yb amplification primer	/5Biosg/CAGGCCGGACTGCGGA*C
2nd round *Alu*Yb amplification primer	AATGATACGGCGACCACCGAGATCTACACTCTTTCCCTACACGACGCTCTTCCGATCTNNNAGTGCTGGGATTACAGGCGTG*A
1st round L1HS amplification primer	/5Biosg/GGGAGATATACCTAATGCTAGATGACAC*A
	/5Biosg/GGGAGATATACCTAATGCTAGATGACAC*G
	/5Biosg/GGGAGATATACCTAATGCTAGATGACAA*G
2nd round L1HS amplification primer	TGCACATGTACCCTAAAACTTAGAGTATAA*T
1st round SVA amplification primer	/5Biosg/AGAATCAGGCAGGGAGGTT*G
2nd round SVA amplification primer	AATGATACGGCGACCACCGAGATCTACACTCTTTCCCTACACGACGCTCTTCCGATCTNNNAGTACMGTCCAGCTTCGGC*T
P7 adaptor amplification primer	CAAGCAGAAGACGGCATACGAGA*T
L1_1 internal primer for validation	GGGAGATATACCTAATGCTAGATGACA
L1_2 internal primer for validation	TGCACATGTACCCTAAAACTTAG
SVA_1 internal primer for validation	AGAATCAGGCAGGGAGGTTG
SVA_2 internal primer for validation	AGTACMGTCCAGCTTCGGCT
/5Biosg/: 5′ Biotin; *: 3′ Phosphorothioate bond; Index1 and Index2: individual specific 6 bp indexes.	

Here, we demonstrate the utility of the ME-Scan protocol in the human genome. Nevertheless, the protocol can be easily extended beyond the human genome by modifying the ME-specific primers. For example, the ME-Scan protocol has been successfully used to study the short interspersed element, Ves, in the bat genus *Myotis* [54]. The high sensitivity and low cost of the ME-Scan protocol makes it an attractive option for studies in non-model organisms.

## Conclusion

The integrated ME-Scan protocol is a cost-effective way to identify novel pMEIs in human genomes. By applying the protocol to three major human mobile element families, we demonstrate the flexibility of the ME-Scan protocol. With a library design instruction, a sequencing protocol, and a computational pipeline for downstream analyses, we present a framework that allows other researchers to easily adapt the ME-Scan protocol to their projects.

## Methods

### Genomic DNA samples

Thirty-six genomic DNA samples from 12 HapMap YRI parent-offspring trios were purchased from Coriell Cell Repositories (https://coriell.org/). Information including individual ID, family ID, and individual relationships is shown in Table S1. DNA samples from three individuals, a mother and her two offspring, were obtained from a previous study [44]. For each individual, DNA samples from four cell types were collected, including CD4^+^ T lymphocytes, iPSCs, NSC, and neurons (referred as “somatic samples” in the following text). Detailed description of these cell lines can be found in the original study [44].

### Library construction and sequencing

The ME-Scan-*Alu*Yb, −L1HS, and -SVA libraries were constructed following the ME-Scan protocol described previously [39, 40] with each ME-specific modifications. The L1HS amplification protocol was adapted from the TIPseq protocol [32, 41]. All the adapters and primers used in this study were synthesized by Integrated DNA Technologies (Coralville, IA, USA) and their sequences are listed in Table 2.

Briefly, 5 μg of each genomic DNA sample in 120 μL TE buffer was randomly fragmented to approximately 1 kb in size using Covaris system (Covaris, Woburn, MA, USA) with the following protocol: duty cycle: 5%; intensity: 3; cycles/burst: 200; time: 15 s. Fragmented samples were concentrated using 120 μL AMPure XP beads (cat. no. A63881, Beckman Coulter, Brea, CA, USA), as previously described [39]. The concentrated DNA fragments and AMPure XP beads (in 50 μl water) were then used to prepare the sequencing libraries using KAPA Library Preparation Kits (cat. no KK8201) or KAPA Hyper Prep Kits with SPRI solution for Illumina (cat. KK8504, KAPA Biosystems, Wilmington, MA, USA).

Following the protocol of KAPA Library Preparation Kit (cat. no KK8201), DNA fragments of the 36 YRI samples were end-repaired and A-tailed on both ends. For the end repaired cleanup, 120 μl PEG/NaCl SPRI Solution was added to 70 μl end repair reaction. For the A-Tailing cleanup, 90 μl PEG/NaCl SPRI Solution was added to 50 μl end repair reaction. The concentration of the A-tailed DNA was measured using a Nanodrop (Thermo Fisher Scientific, Wilmington, DE, USA), and these A-tailed DNA fragments were then ligated with a different index-adapter, providing each individual a unique downstream identity. The concentration of ligated DNA from each sample was determined using Nanodrop. For the 36 YRI samples, 14 and 22 samples were pooled into two different libraries with equal concentration for each sample. Sequencing libraries of the 12 somatic samples were constructed following the protocol of KAPA Hyper Prep Kit (cat. No KK8504). The concentration of ligated DNA from each sample was determined using Nanodrop, and the samples were pooled into a single library with equal concentration. The following steps were performed using the pooled libraries.

For each ME family, two rounds of ME-specific amplification were conducted. The detailed amplification conditions and protocols are shown in Table 3 and Table 4. For the first round, *Alu*Yb and SVA libraries were amplified using a standard PCR protocol: initial denaturation at 98 °C for 45 s, followed by the thermocycling conditions of 98 °C for 15 s, 65 °C for 30 s, and 72 °C for 30 s, and a final extension at 72 °C for 1 min. L1HS libraries were amplified using a step-down protocol (Table 4), similar to the TIPSeq protocol [32]. For L1HS and SVA amplified PCR products, size selection was performed using 0.7X of PEG/NaCl SPRI Solution. Biotinylated ME-enriched DNA fragments were then magnetically separated from other genomic DNA fragments using 5 μl Dynabeads^R^ M-270 Streptavidin (cat. no. 65305, Invitrogen, Life Technologies, Oslo, Norway) following the manufacturer’s protocol. PCR products from the second amplification were electrophoresed at 120 V/90 min for SVA; 100 V/120 min for *Alu*Yb and L1HS on a 2% NuSieve^R^ GTG^R^ Agarose gel (cat. no. 50080, Lonza, Rockland, Maine, USA). Fragments around 500 bp were size selected and purified using Wizard SV Gel and PCR Clean-up system (cat. no. A9281, Promega, Madison, WI, USA). Before the libraries were sequenced, their fragment size and concentration were quantified using Bioanalyzer and quantitative PCR by the RUCDR Infinite Biologics (Piscataway, NJ, USA).

**Table 3:** ME-Scan amplification conditions

	First amplification			Second amplification		
	*Alu*Yb (5 cycles)	L1HS*	SVA (10 cycles)	*Alu*Yb (20 cycles)	L1HS (12 cycles)	SVA (12 cycles)
PCR grade water	As needed			As needed		
2X KAPA HiFi HS RM	25 μl			25 μl		37.5 μl
Adapter primer (P7)^+^	2.5 μl			2.5 μl		3.75 μl
ME-specific primer ^+^	2.5 μl			2.5 μl		3.75 μl
DNA	360 ng	100 ng	200 ng	16 μl	2 μl	24 μl
Total	50 μl			50 μl		75 μl

* follow step-down PCR thermocycling conditions in Table 4

^+^ primers are shown in Table 2 with 10 μM concentration

**Table 4:** Step-down PCR thermocycling condition for L1HS amplification

95 °C	5 min	
95 °C	1 min	Repeat 5 cycles
72 °C	1 min	
72 °C	5 min	
95 °C	1 min	Repeat 5 cycles
68 °C	1 min	
72 °C	5 min	
95 °C	45 s	Repeat 15 cycles
64 °C	1 min	
72 °C	5 min	
72 °C	15 min	
4 °C	Hold	

For the 12 somatic samples, after ME-specific amplifications, purified PCR products from *Alu*Yb-, L1HS-, and SVA-sequencing libraries were pooled into a single library with a 1:4:4 ratio. The different ratio was applied to increase the depth of coverage for L1HS and SVA elements. All the libraries were sequenced using the Illumina HiSeq 2000 with 100PE format at RUCDR Infinite Biologics. The sequencing data have been deposited to SRA under project number SRP129897.

### Computational analysis

The computational analysis pipeline was comprised of bash and python codes. The codes are available at https://github.com/JXing-Lab/ME-SCAN_2018 and the overall workflow is shown in Fig. S1. Briefly, ncbi-blast-2.2.28+ [55] was used to compare the ME sequence (*Alu*Yb, L1HS, or SVA) in each ME Read to the corresponding ME consensus sequence to generate the BLAST bit-score, by running the command “blastn -task blastn-short -db MEI_primer.fasta -query read1.fasta -outfmt 6 -out read1_MEI_blast.out”. BWA-MEM (ver. 0.7.5a) [56] was used to map the Flanking Read against the human reference genome (hg19), by running the command “bwa mem hg19.fa read2.fastq > read2_BB.sam”. The default parameters of BWA-MEM are: matching score:1, mismatch penalty:4, gap open penalty:6, gap extension penalty:1, and clipping penalty:5. Samtools-1.1 [57] was used to count the number of Flanking Reads that were mapped to the human reference genome in each individual. BEDTools (Ver. 2.16.2) [58] was used to cluster all mapped reads in a region and to generate a list of representative insertion loci. To obtain high quality loci, TPM and UR were calculated for each locus using customized python and bash codes. Results from all applications were integrated into the current pipeline.

Known polymorphic loci were acquired from the Database of Retrotransposon Insertion Polymorphisms (dbRIP, [59]), HuRef genome [6], and the 1000 Genomes Project [4, 7]. For the sensitivity analysis and TPM/UR cutoff selection, presumed fixed reference MEIs are defined as MEIs that are present in the reference genome and are not reported as polymorphic MEIs in previous studies [4, 6, 7, 59]. Gene annotation and chromatin state profiles from nine cell lines were obtained from GENCODE (Release 19) and ChromHMM [42], respectively. For each chromatin state, the normalized number of MEIs (number of insertions divided by total number of locations in each state) was calculated.

### Genotyping PCR for validation

PCR validation was performed for eight pMEI loci from the YRI samples (Table S5) and two loci from the somatic samples (Table S7). For *Alu*Yb loci, only one pair of primer was needed for validation. For L1 and SVA, an internal primer was needed to validate the presence of the insertion. The PCR reactions were performed as previously described [6, 40].

The PCRs were performed using One Taq hot start DNA polymerase with GC buffer (cat. no. M0481, New England Biolabs, Ipswich, MA, USA). The reactions were set up in 25 μl volume according to the manufacturer’s standard protocol. In each reaction, 100 nanograms of genomic DNA from the original samples were used as template. The thermocycling condition was: an initial denaturation at 94 °C for 30 s, followed by 30 cycles of 94 °C for 30 s, a locus-specific annealing temperature (Table S5, S7) for 1 min, and 68 °C for 3 min, followed by a final extension at 68 °C for 3 min. The PCR products were electrophoresed at 300 V for 25 min on a 1.5% GenePure LE Agarose gel (cat. no. E-3120-500, BioExpress, Kaysville, UT, USA). Sanger sequencing was performed by Genewiz (South Plainfield, NJ, USA).

## Supplementary information

**Additional file 1.** List of pMEIs in the 36 YRI samples. The file is provided in the Variant Call Format (VCF, https://samtools.github.io/hts-specs/VCFv4.2.pdf).

**Additional file 2.** List of pMEIs in the three somatic samples. The file is provided in the Variant Call Format (VCF, https://samtools.github.io/hts-specs/VCFv4.2.pdf).

**Additional file 3: Figure S1.** Computational pipeline for ME-Scan analysis.

**Additional file 4: Figure S2.** Distribution of ME Read BLAST bit-scores in RepeatMasker annotated MEs in the human reference genome. A) *Alu*Yb; B) L1HS; C) SVA. Cutoffs used in this study are labeled with arrows for each ME type.

**Additional file 5: Figure S3.** Potential functional impact of pMEIs. A) functional annotation; B) abundance of pMEIs in different chromatin states. Chromatin state profiles (Y-axis) from nine cell lines (X-axis) were obtained from ChromHMM [42]. For each chromatin state, the normalized number of pMEIs is shown. Chromatin States: 1 - Active Promoter, 2 - Weak Promoter, 3 - Inactive/poised Promoter, 4 - Strong enhancer, 5 - Strong enhancer, 6 - Weak/poised enhancer, 7 - Weak/poised enhancer, 8 – Insulator, 9 - Transcriptional transition, 10 - Transcriptional elongation, 11 - Weak transcribed, 12 - Polycomb-repressed, 13 - Heterochromatin; low signal, 14 - Repetitive/CNV, 15 - Repetitive/CNV.

**Additional file 6: Figure S4.** Locus-specific PCR validation. A) Alu_CDS1; B) L1_CDS2, L1_CDS4, L1_CDS5, L1_CDS7; C) Sequence of the Alu_CDS1 locus. Each individual ID is labelled on the top of the lane. Ladder, 100 bp ladder. The main ladder bands (500 bps, 1000 bps) are labelled. The expected empty allele (i.e. no insertion) size is labelled on the top of the lane. For L1 loci, three PCR reactions were performed. Left panel: E: outside forward + reverse primer, I: internal L1_1 + outside forward primer; right panel: internal L1_2 + outside forward primer. The expected internal + external size is around 500 bps although it varies because of the variable poly(A) length of the L1 insertion. The expected internal + outside primer amplification product is indicated by black arrow. Because we were unable to amplify the full insertion allele by the external primers, we did not validate both ends of the L1 insertion by Sanger sequencing. For the Sanger sequencing result Alu_CDS1, the *Alu*Y insertion is highlighted in green, the target site duplications are in red, and the potential endonuclease cutting site is underlined.

**Additional file 7: Table S1.** Number of passed filter reads for YRI samples.

**Additional file 8: Table S2.** Candidate *de novo* pMEIs in YRI families.

**Additional file 9: Table S3.** Inheritance error rates for pMEIs in YRI families.

**Additional file 10: Table S4.** Inheritance rates for pMEIs in YRI families.

**Additional file 11: Table S5.** pMEIs overlapping CDS regions in YRI families.

**Additional file 12: Table S6.** Number of passed filter reads for somatic samples.

**Additional file 13: Table S7.** SVA somatic insertion candidates.

## Data Availability

The final datasets supporting the conclusions of this article are included within the article and its additional files. The raw sequencing data is available in the NIH Sequence Read Archive (SRA) under project number PRJNA430450 (https://www.ncbi.nlm.nih.gov/bioproject/PRJNA430450/). The ME-Scan computational pipeline are available at https://github.com/JXing-Lab/ME-SCAN_2018.

## Abbreviations

CDS: Coding sequence
LTR: Long terminal repeat
ME: Mobile element
MEI: Mobile element insertion
ME-Scan: Mobile element scanning
pMEI: polymorphic mobile element insertion
TPM: Tags per million
UR: Unique read
UTR: Untranslated region
VNTR: Variable number of tandem repeat
