# Supplementary figures and images for: Integrated Mobile Element Scanning (ME-Scan) method for identifying multiple types of polymorphic mobile element insertions
Source: Mob DNA. 2020 Feb 22;11:12. doi: 10.1186/s13100-020-00207-x (PMC7035633; doi:10.1186/s13100-020-00207-x)

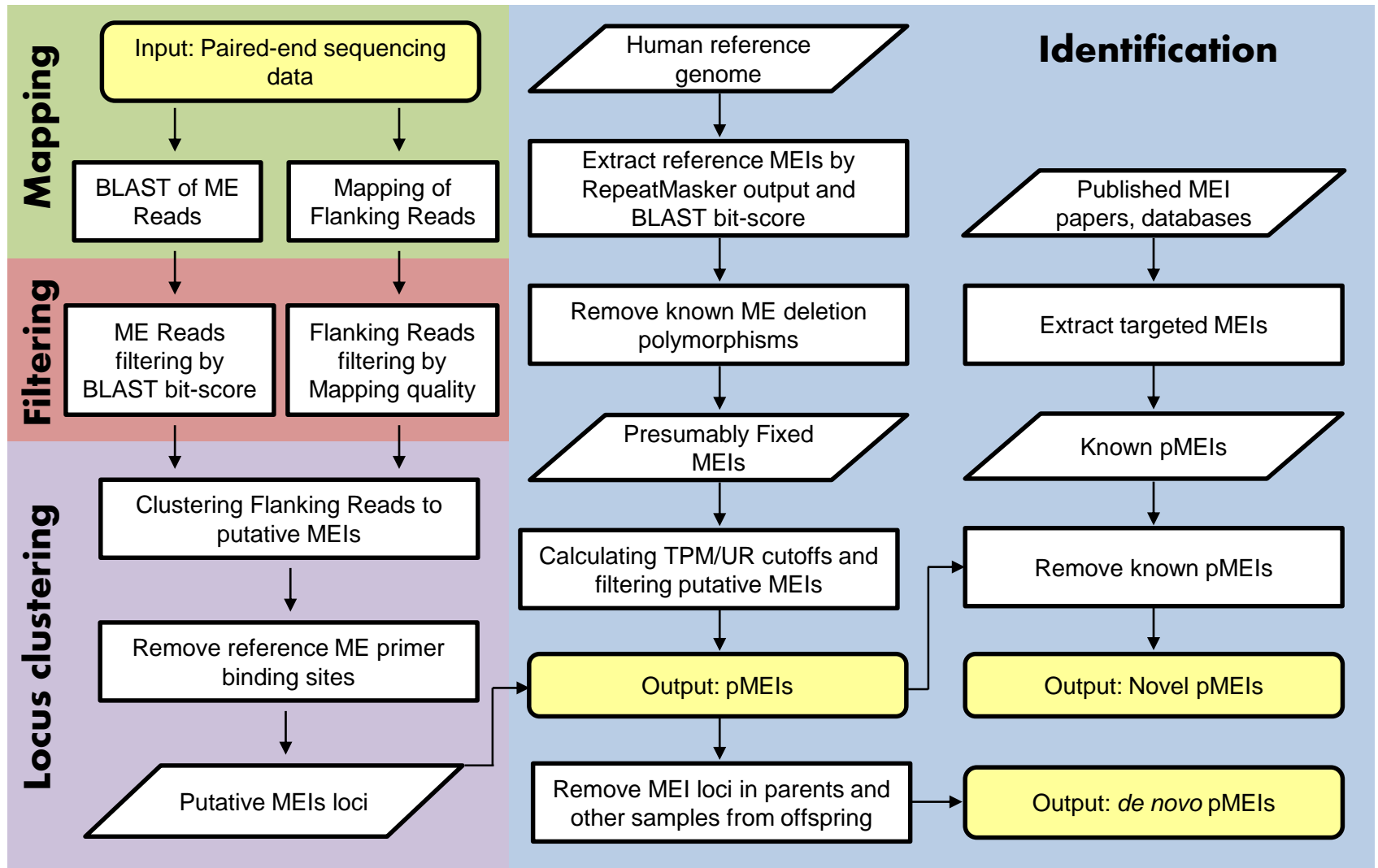

Supplement: Supplementary file 3 — Additional file 3: Figure S1. Computational pipeline for ME-Scan analysis. [file 13100_2020_207_MOESM3_ESM.pdf]

## A) Alu

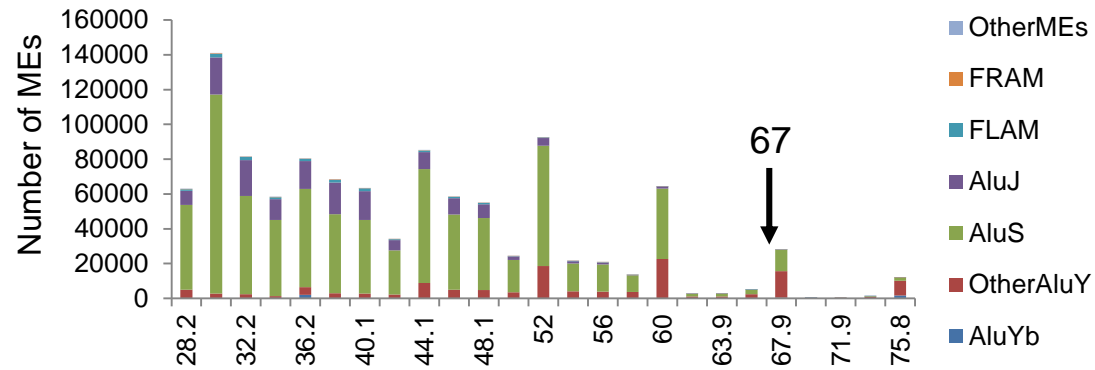

## B) L1

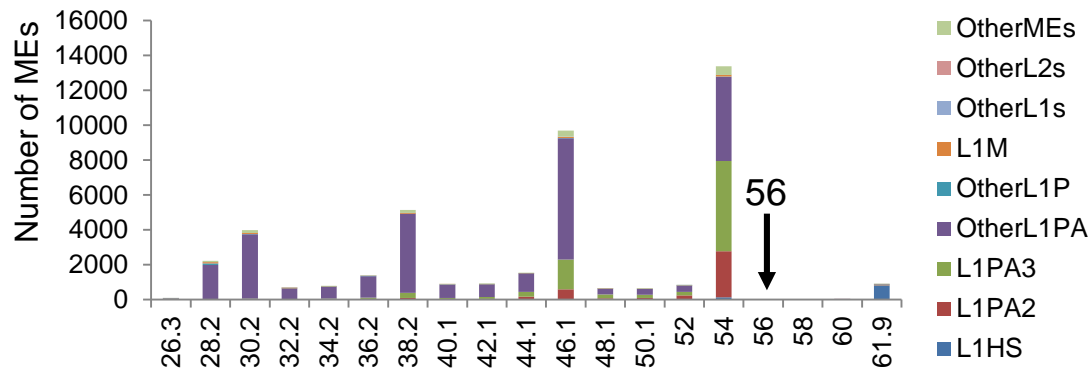

## C) SVA

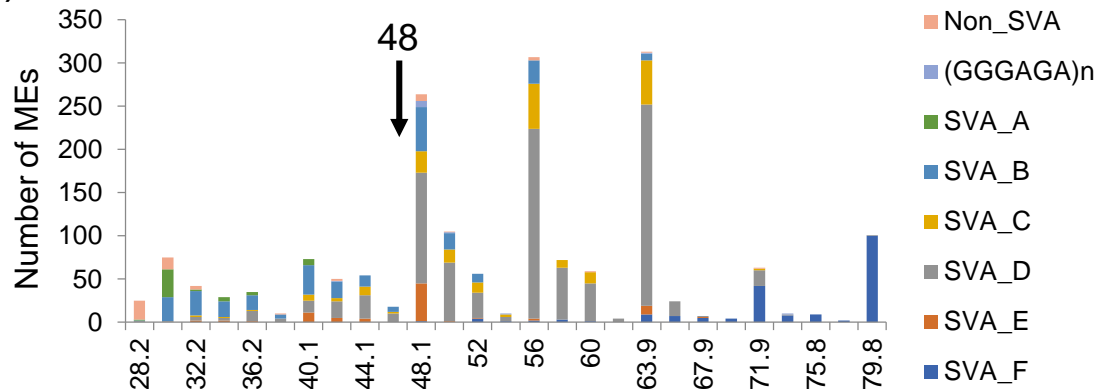

Supplement: Supplementary file 4 — Additional file 4: Figure S2. Distribution of ME Read BLAST bit-scores in RepeatMasker annotated MEs in the human reference genome. A) AluYb; B) L1HS; C) SVA. Cutoffs used in this study are labeled with arrows for each ME type. [file 13100_2020_207_MOESM4_ESM.pdf]

# A) Alu

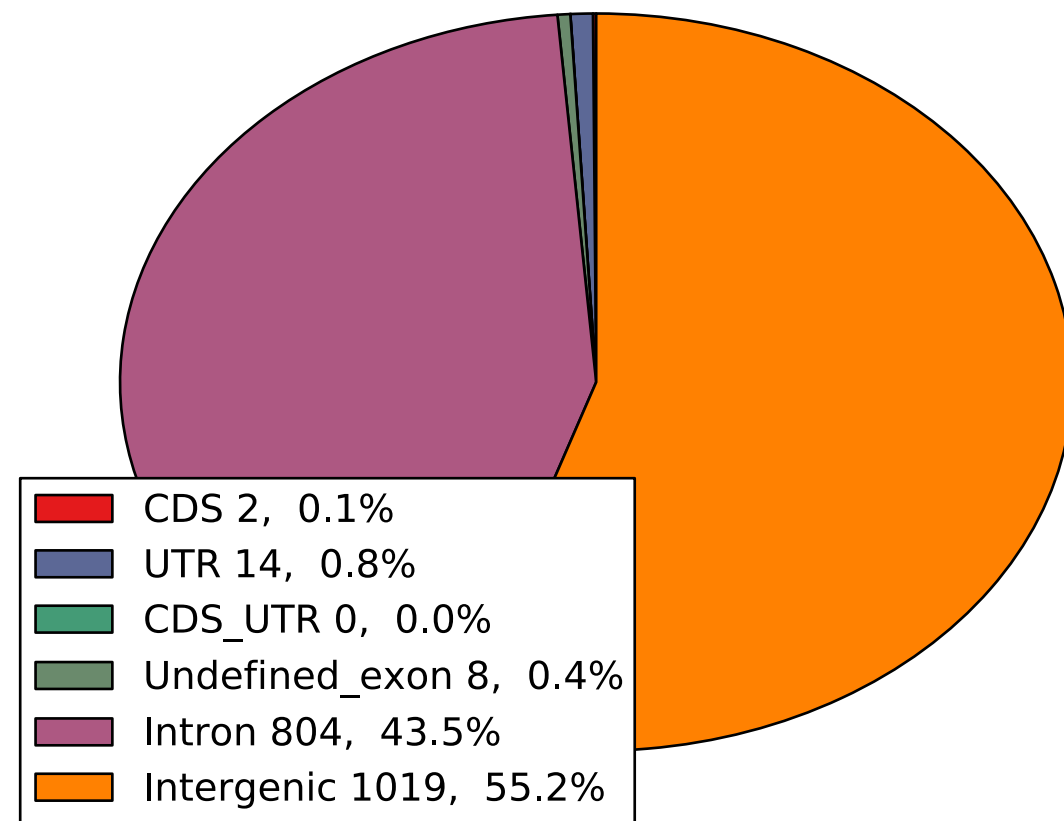

# L1HS

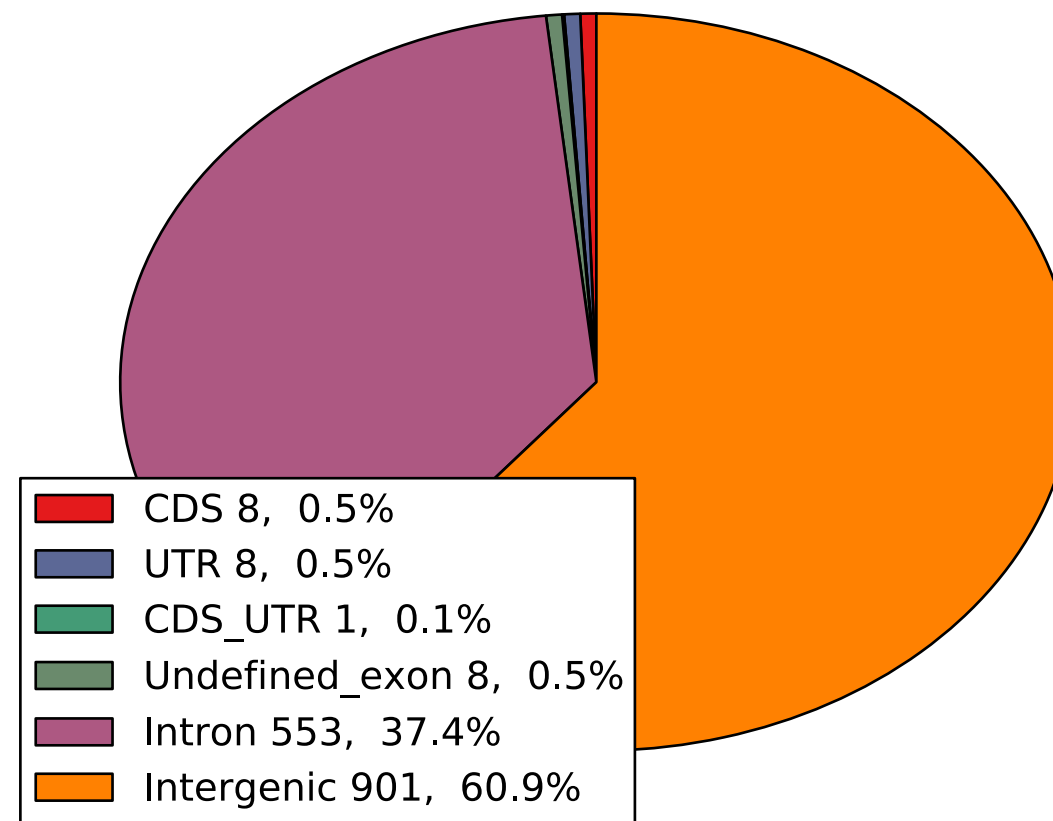

# SVA

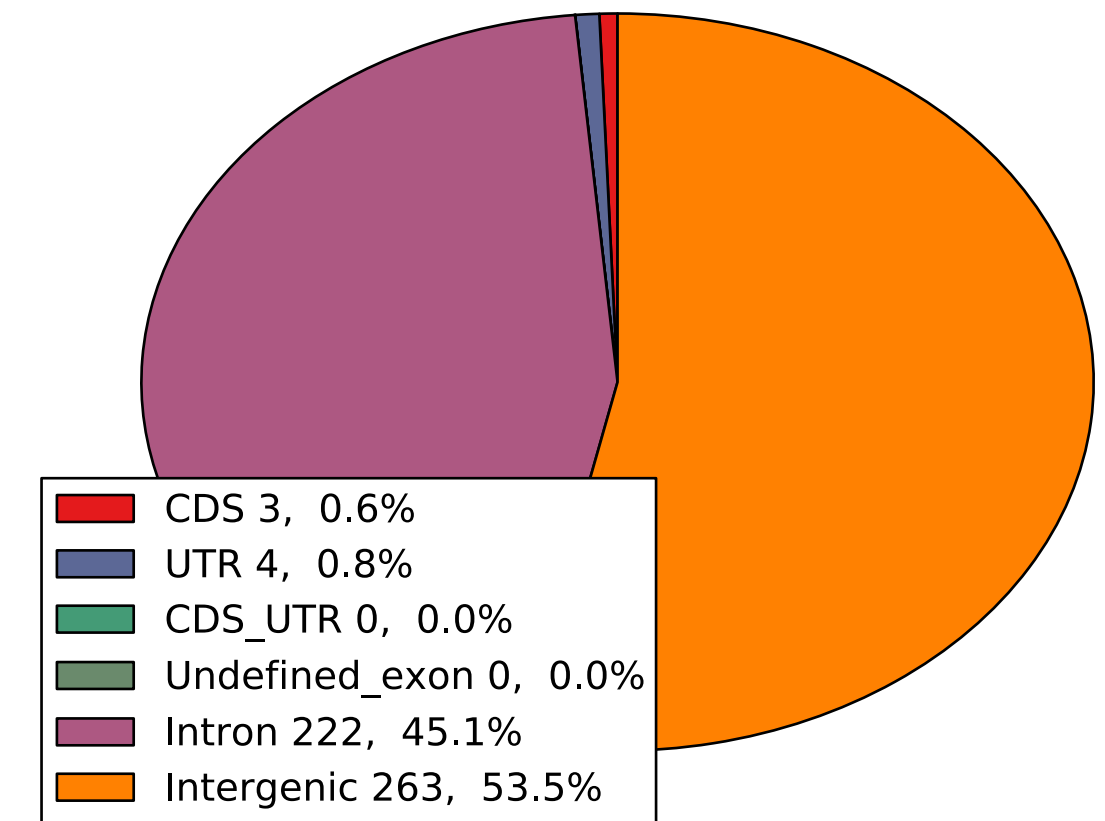

# B) Alu

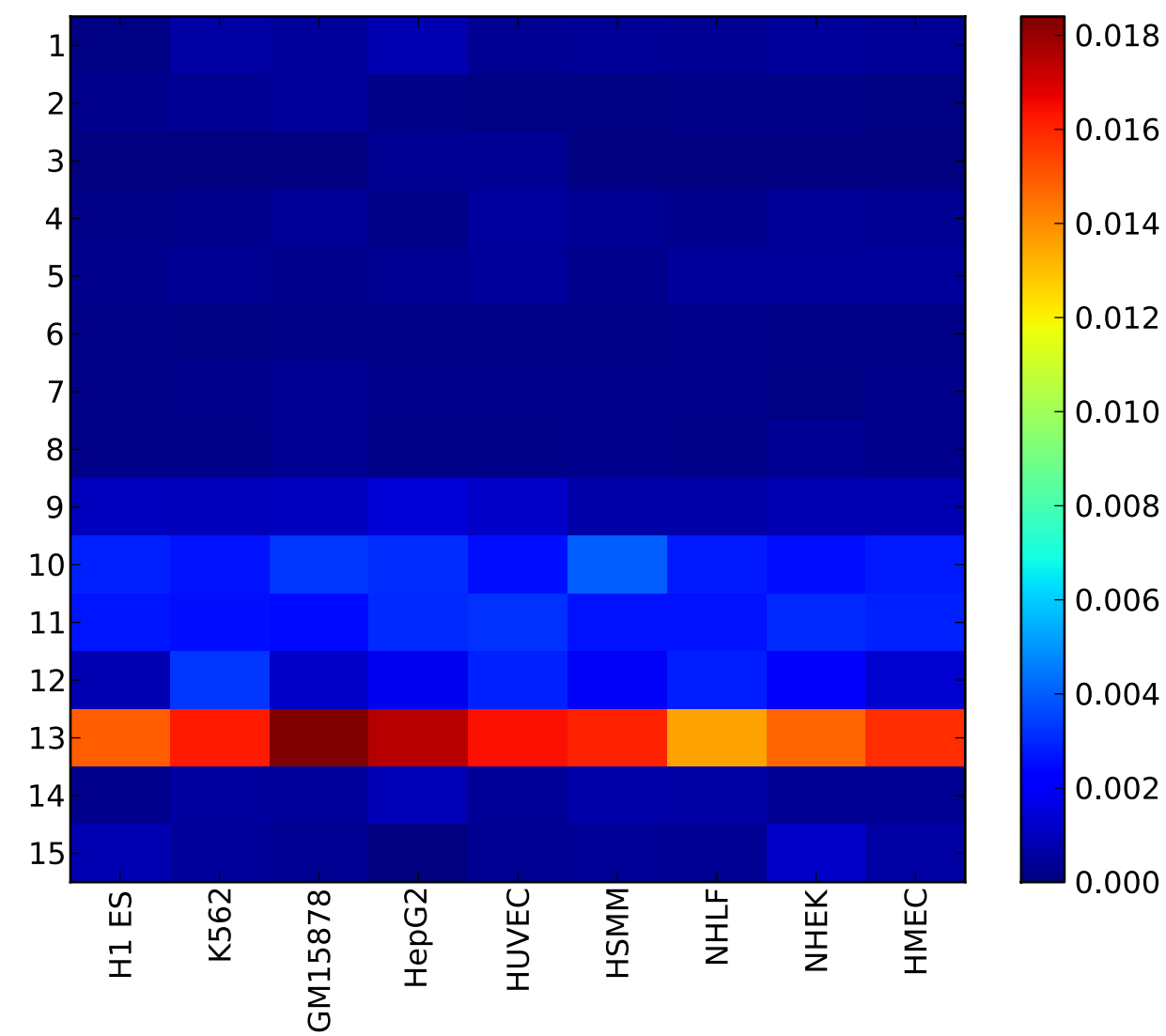

# L1HS

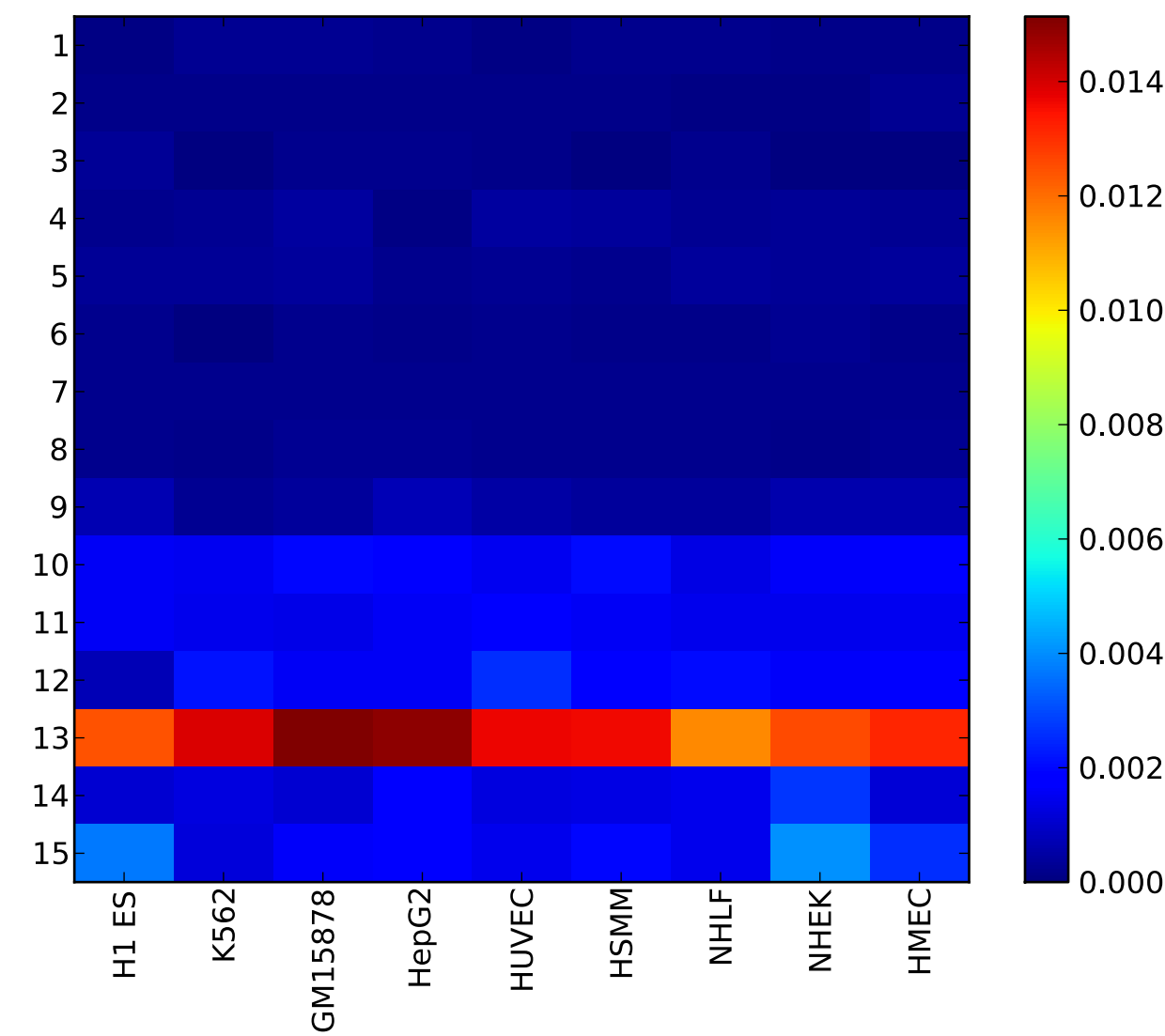

# SVA

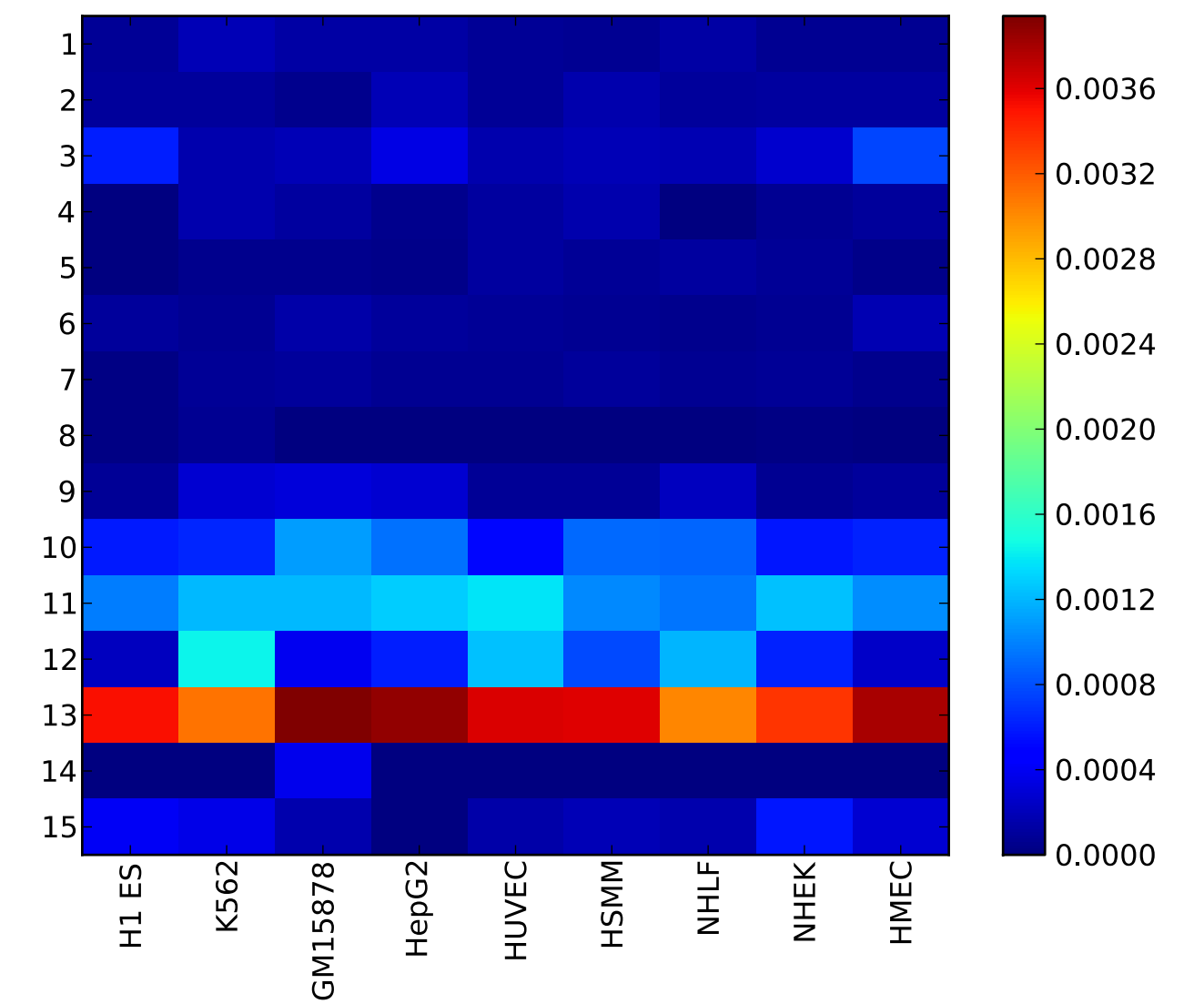

Supplement: Supplementary file 5 — Additional file 5: Figure S3. Potential functional impact of pMEIs. A) functional annotation; B) abundance of pMEIs in different chromatin states. Chromatin state profiles (Y-axis) from nine cell lines (X-axis) were obtained from ChromHMM [42]. For each chromatin state, the normalized number of pMEIs is shown. Chromatin States: 1 - Active Promoter, 2 - Weak Promoter, 3 - Inactive/poised Promoter, 4 - Strong enhancer, 5 - Strong enhancer, 6 - Weak/poised enhancer, 7 - Weak/poised enhancer, 8 – Insulator, 9 - Transcriptional transition, 10 - Transcriptional elongation, 11 - Weak transcribed, 12 - Polycomb-repressed, 13 - Heterochromatin; low signal, 14 - Repetitive/CNV, 15 - Repetitive/CNV. [file 13100_2020_207_MOESM5_ESM.pdf]
