# Supplementary material for: Integrated Mobile Element Scanning (ME-Scan) method for identifying multiple types of polymorphic mobile element insertions
Source: Mob DNA. 2020 Feb 22;11:12. doi: 10.1186/s13100-020-00207-x (PMC7035633; doi:10.1186/s13100-020-00207-x)
Supplement: Supplementary file 6 — Additional file 6: Figure S4. Locus-specific PCR validation. A) Alu_CDS1; B) L1_CDS2, L1_CDS4, L1_CDS5, L1_CDS7; C) Sequence of the Alu_CDS1 locus. Each individual ID is labelled on the top of the lane. Ladder, 100 bp ladder. The main ladder bands (500 bps, 1000 bps) are labelled. The expected empty allele (i.e. no insertion) size is labelled on the top of the lane. For L1 loci, three PCR reactions were performed. Left panel: E: outside forward + reverse primer, I: internal L1_1 + outside forward primer; right panel: internal L1_2 + outside forward primer. The expected internal + external size is around 500 bps although it varies because of the variable poly(A) length of the L1 insertion. The expected internal + outside primer amplification product is indicated by black arrow. Because we were unable to amplify the full insertion allele by the external primers, we did not validate both ends of the L1 insertion by Sanger sequencing. For the Sanger sequencing result Alu_CDS1, the AluY insertion is highlighted in green, the target site duplications are in red, and the potential endonuclease cutting site is underlined. [file 13100_2020_207_MOESM6_ESM.pdf]

A B

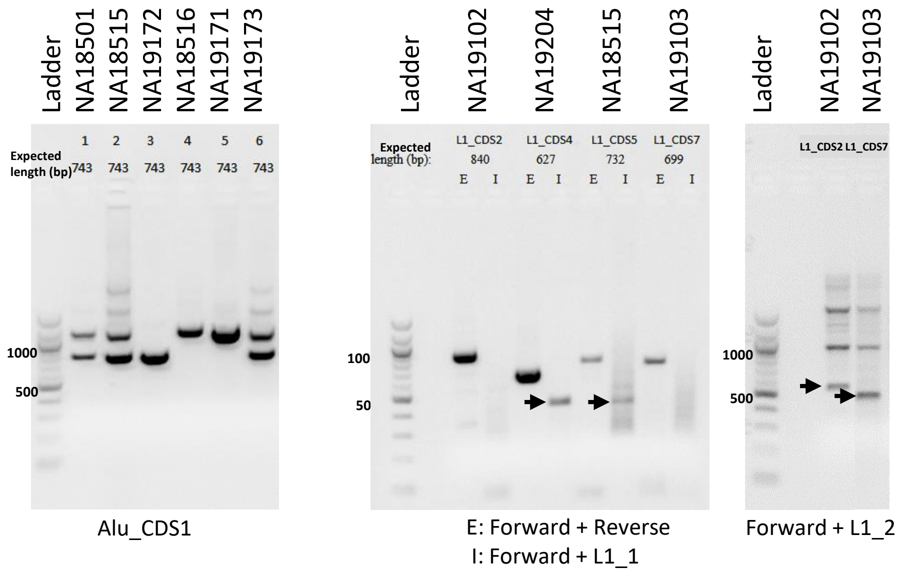

C

Alu_CDS1: chr8: 59498595 - 59499253

GCTTCAGAATGNGGTTTACGTTATTCTGGATTTTATTTCTGAGTCCAGTGGCCTAATC
TCCACATTCGTTTTCTTCAAAATCCTTGTACTTAAGCAATGACTATCCTCTCCTTGAA
ATGGTTCTAGCCCTTTCCAAGATGTAAGGATGAGATGACCCTTTGATTATGAGTTCAT
TTTTATCTTAAATTAATTTCTCTAATGAGGAATTAAAAATACCCAGTCCGGCTTTTCT
ACCTGATACAAATGCCACAAGTATACTGTACCCTGGCATAAAGCAGTGTTAGCCATTG
GCAAAACCTCTTTTATCAGCAAATAGTGTTTTTCTTTGTAAGTTTTATTGTTTATTTC
AAAATTAACAATATTGTTTATTTCAAAATTCCAATTTACCTAGTATTATCTGTTTTAA
AAAGGATAATATCGGCCGGGCGCGGTGGCTCACGCCTGTAATCCCAGCACTTTGGGAG
GCCGAGGCGGGTGGATCATGAGGTCAGGAGATCGAGACCATCCTGGCTAACAAGGTGA
AACCCCGTCTCTACTAAAAATACAAAAAATTAGCCGGGCGCGGTGGCGGGCGCCTGTA
GTCCCAGCTACTCGGGAGGCTGAGGCAGGAGAATGGCGTGAACCCGGGAAGCGGAGCT
TGCAGTGAGCCGAGATTGCGCCACTGCAGTCCGCAGTCCGGCCTGGGCGACAGAGCGA
GACTCCGTCTCAAAAAAAAAAAAAAAAAAAAAAAGGATAATATCTACCTGGGCTAAAA
GCAGTGTCACATACAATCCCTGAATGGCATGGAATCTGGTGCATTAAGGTGGCCGTTG
TGAGGTCCCAAATATTCACTGTGCCTTCTTTGGTGCCGGAAACTAACAGTGTGCTTGC
AGCATTTAAACTGATTGTATCTACCTAAGGAAGAAAACACATTGATACATATTCCATT
AGATTTATGGTCATTAGCTATGTTTCTCTAGTCNGGNTCAAATATGTAGTCA
